# Specific Engineered G Protein Coupling to Histamine Receptors Revealed from Cellular Assay Experiments and Accelerated Molecular Dynamics Simulations

**DOI:** 10.3390/ijms221810047

**Published:** 2021-09-17

**Authors:** Carina Höring, Marcus Conrad, Christian A. Söldner, Jinan Wang, Heinrich Sticht, Andrea Strasser, Yinglong Miao

**Affiliations:** 1 Institute of Pharmacy, Faculty of Chemistry and Pharmacy, University of Regensburg, 93040 Regensburg, Germany; andrea.strasser@ur.de; 2 Bioinformatik, Institut für Biochemie, Friedrich-Alexander-Universität Erlangen-Nürnberg (FAU), Fahrstraße 17, 91054 Erlangen, Germany; mar.conrad@fau.de (M.C.); christian.soeldner@gmx.de (C.A.S.); heinrich.sticht@fau.de (H.S.); 3 Department of Computational Biology and Molecular Biosciences, University of Kansas, Lawrence, KS 66047, USA; jawang@ku.edu; 4 Erlangen National High Performance Computing Center (NHR@FAU), Friedrich-Alexander-University Erlangen-Nürnberg (FAU), 91058 Erlangen, Germany

**Keywords:** GPCR–G protein coupling profiles, Gaussian accelerated molecular dynamics (GaMD), split-luciferase complementation assay, histamine signaling, histamine H_2_ receptor, histamine H_4_ receptor, engineered G proteins

## Abstract

G protein-coupled receptors (GPCRs) are targets of extracellular stimuli and hence occupy a key position in drug discovery. By specific and not yet fully elucidated coupling profiles with α subunits of distinct G protein families, they regulate cellular responses. The histamine H_2_ and H_4_ receptors (H_2_R and H_4_R) are prominent members of Gs- and Gi-coupled GPCRs. Nevertheless, promiscuous G protein and selective Gi signaling have been reported for the H_2_R and H_4_R, respectively, the molecular mechanism of which remained unclear. Using a combination of cellular experimental assays and Gaussian accelerated molecular dynamics (GaMD) simulations, we investigated the coupling profiles of the H_2_R and H_4_R to engineered mini-G proteins (mG). We obtained coupling profiles of the mGs, mGsi, or mGsq proteins to the H_2_R and H_4_R from the mini-G protein recruitment assays using HEK293T cells. Compared to H_2_R–mGs expressing cells, histamine responses were weaker (pEC_50_, E_max_) for H_2_R–mGsi and –mGsq. By contrast, the H_4_R selectively bound to mGsi. Similarly, in all-atom GaMD simulations, we observed a preferential binding of H_2_R to mGs and H_4_R to mGsi revealed by the structural flexibility and free energy landscapes of the complexes. Although the mG α5 helices were consistently located within the HR binding cavity, alternative binding orientations were detected in the complexes. Due to the specific residue interactions, all mG α5 helices of the H_2_R complexes adopted the Gs-like orientation toward the receptor transmembrane (TM) 6 domain, whereas in H_4_R complexes, only mGsi was in the Gi-like orientation toward TM2, which was in agreement with Gs- and Gi-coupled GPCRs structures resolved by X-ray/cryo-EM. These cellular and molecular insights support (patho)physiological profiles of the histamine receptors, especially the hitherto little studied H_2_R function in the brain, as well as of the pharmacological potential of H_4_R selective drugs.

## 1. Introduction

In the human body, the neurotransmitter histamine interacts with four subtypes of histamine receptors (H_1-4_R) that are all classified as rhodopsin-like class A G protein-coupled receptors (GPCRs) [1]. As the largest membrane protein superfamily [2,3], GPCRs have been intensively studied as important drug targets over the past decades, leading to more than 30% of approved drugs binding to GPCRs [4]. In the 1970s, H_2_R antagonists, such as cimetidine and ranitidine, were among the blockbuster drugs on the market, reducing gastric acid secretion [5]. In addition to the expression in gastric parietal cells, the H_2_R is also widely found in smooth muscle cells, chondrocytes, endothelial and epithelial cells, dendritic cells, and macrophages as well as T and B cells [1]. Currently, the H_2_R function in the central nervous system (CNS) is investigated with CNS-penetrating agonists [6,7]. In contrast, no substance has yet been approved for medical treatment that selectively binds to the Gi-coupled H_4_R. The involvement of the H_4_R in allergic and inflammatory processes is undisputed [8,9,10] due to its expression being described mainly in immune cells, mast cells, and eosinophils [11,12,13]. Particularly, the latter expression has raised the H_4_R as a potential target for the treatment of atopic dermatitis [14,15], and currently, the first antagonist, ZPL-3893787, is being considered for use in patients [16].

One of the key events in GPCR signaling is the activation of heterotrimeric G proteins consisting of the α, β, and γ subunits [17,18,19]. Today, more than 800 GPCRs and 16 G proteins have been identified in the human genome. There is evidence that GPCRs interact with multiple intracellular G proteins, giving unique coupling profiles with multidimensional cellular effects [20,21,22,23,24]. In the 1990s, the human H_2_R was cloned and demonstrated to couple to Gs due to the increasing cAMP level upon receptor activation [25]. Kühn et al. reported an H_2_R interaction with Gq by immunoprecipitation of H_2_R-Gq assemblies and by inositol phosphate accumulation when the H_2_R was co-expressed with Gq family members Gαq, Gα11, Gα14, and Gα15 [26]. Around the millennium, the human H_4_R was cloned by several groups [27,28,29,30,31,32,33], and Gi coupling has been proven by the inhibition of forskolin-induced cAMP accumulation. In addition, an increase in intracellular Ca^2+^ has been reported in eosinophils [34], monocytes [35], and mast cells [11,36] upon H_4_R activation, which was initially discussed as a βγ signal activating phospholipase C [11] (PLC) but later was identified as an H_1_R-mediated response [36]. In terms of Gα subtype selectivity, a preference of Gαi_2_ over Gαi_1_, Gαi_3_, and Gαo proteins was reported for the H_4_R in a [^35^S]GTPγS binding assay, in which the amount of the non-hydrolyzable guanine nucleotide analog bound to the H_4_R-Gα complex was quantified [37]. Such proximal assay readouts appear advantageous with respect to signal amplification along the signaling cascade by different second messengers and thus misinterpretation of the intracellular effects.

Nowadays, several assay techniques have facilitated the analysis of GPCR G protein interactions, such as BRET-based heterotrimeric G protein biosensors either detecting the dissociation of the heteromer into an α monomer and a βγ dimer [38] or the dissociation of the βγ dimer from the receptor [39] as well as the BRET-based effector membrane translocation assay (EMTA) monitoring the activation of Gα proteins by the recruitment of specific G protein effector molecules [40]. Another prominent technique to study GPCR–G protein interactions is the application of chimeric G proteins [41], which essentially consists of the α5 helix of a G protein subtype and a G protein core. The α5 helix accounts for more than 70% of the contact area between a GPCR and G protein and thus dictates successful binding [39,42,43]. In contrast, the G protein core is used to redirect signals into a single readout. For instance, Inoue et al. applied 11 chimeric G proteins from all major G protein families to demonstrate the coupling behavior of 148 GPCRs by redirecting all signals into TGF-α triggering [44]. A similar approach has been used in the development of genetically engineered mini-G (mG) proteins consisting of the GTPase domain of Gαs and the α5 helices of G proteins of the four major G protein families Gs, Gi/o, Gq/11, and G12/13 [45,46,47]. Combined with BRET or split-luciferase complementation techniques, the mini-G proteins are suitable for ligand characterization in cell-based assays [47,48,49,50,51,52]. Moreover, these proteins were originally designed to stabilize GPCRs in their active state and thus to enable the elucidation of GPCR–G protein complexes by X-ray and cryo-electron microscopy (cryo-EM), which has been achieved for the adenosine A_2A_ (A_2A_R) [53], the dopamine D_1_ (D_1_R) [54], the GPR52 [55], the serotonin 5-HT_1B_ (5-HT_1B_R) [56], and 5-HT_2A_ (5-HT_2A_R) [57] receptors so far. Recent years have seen remarkable advances in the structural determination of GPCR–G protein complexes [58,59]. In 2020 alone, new structures of 34 GPCR–Gs complexes, 19 GPCR-Gi/o complexes, and one GPCR-Gq/11 complex were published (www.gpcrdb.org, access date 8 October 2021 [58]), providing valuable atomic-level insights into the binding interface of GPCRs with G proteins from different major families. Another milestone in histamine receptor research was the resolution of the first active structure of the histamine H_1_ receptor in complex with Gq in 2021 [60]. Nevertheless, static structures alone do not allow us to map the dynamics of a GPCR–G protein interaction, and only complexes of primary coupling GPCRs and G proteins are available so far. Thus, the dynamic mechanism of specific GPCR–G protein interactions remains poorly understood. To fill this gap, computational approaches have been developed to model the dynamic GPCR–G protein interactions [61,62]. For instance, Flock et al. provided a bioinformatics approach to determine a selectivity barcode (patterns of amino acids) of GPCR−G protein coupling [42]. More commonly, molecular dynamics (MD) simulations have been used to explore the conformational changes and free energy landscapes of GPCR–G protein interactions, ideally combined with complementary experiments [63,64,65]. However, conventional MD (cMD) simulations often suffer from insufficient sampling of GPCR–G protein interactions due to limited simulation timescales. Thus, enhanced sampling methods have been applied to improve the simulations of GPCR–G protein interactions [62,66]. Among these methods, Gaussian accelerated molecular dynamics (GaMD) is a robust method that allows for unconstrained enhanced sampling and free energy calculations of large biomolecules [67,68,69,70]. GaMD has been applied to successfully simulate protein folding [68,71], protein-ligand binding and unbinding [67,68,72], GPCR activation [72], and binding to a G protein mimetic nanobody [73]. In the latter study, the nanobody binding pathway to the muscarinic acetylcholine M_2_ (M_2_R) receptor has been investigated, demonstrating that the intracellular loops play a key role in nanobody recognition and binding [73]. Moreover, GaMD has been used to identify the coupling mechanisms of adenosine A_1_ (A_1_R) and A_2A_ (A_2A_R) receptors to Gs and Gi proteins [74]. Protein flexibility and complementary residue interactions at the protein interface have revealed that the A_1_R preferred Gi coupling and the A_2A_R coupled to Gs and Gi [74].

In the present study, we have combined cellular assay experiments and GaMD-enhanced sampling simulations to investigate coupling profiles of the H_2_R- and H_4_R-G protein complexes. Three types of engineered G proteins, mGs, mGsi, and mGsq, were used to characterize the coupling profiles of each receptor. We have explored the functional responses of both receptors upon activation by the endogenous ligand histamine using a recently published mini-G protein recruitment assay [52]. Furthermore, we performed all-atom GaMD simulations on the H_2_R and H_4_R in complex with mGs, mGsi, and mGsq in explicit lipids and solvent. In all six systems, the HRs were bound to the endogenous ligand histamine. The GaMD simulations have allowed us to characterize the structural flexibility and low-energy conformations of the HRs in complex with the mGs, mGsi, and mGsq proteins. The combination of cellular assay experiments and GaMD simulations has provided important mechanistic insights into the selective coupling of engineered G proteins to the HRs.

## 2. Results and Discussion

### 2.1. Functional Characterization of Histamine at H_2_R– and H_4_R–Mini-G Protein Complexes

**Figure 1:**
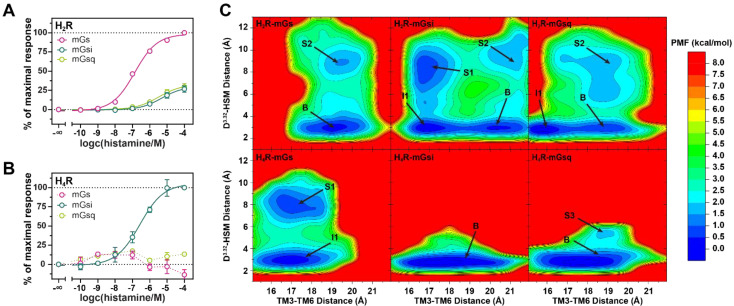
Concentration–response curves of histamine obtained in a split-Nanoluciferase based mini-G protein recruitment assay using HEK293T cells transiently expressing either the H_2_R (**A**) or the H_4_R (**B**) in combination with mGs, mGsi, or mGsq. Presented data are of three independent experiments (*N* = 3), each performed in triplicate. (**C**) Free energy profiles of GaMD simulations with complexes containing either the H_2_R or the H_4_R in combination with mGs, mGsi, or mGsq. Distances of the D^3.32^ (Cγ atom) and the amino group of histamine (Nα atom) as well as of the intracellular ends of TM3 and TM6 were used as reaction coordinates. TM3–TM6 distances were calculated using Cα, C, and N atoms of R^3.50^ and E/A^6.30^. For each system, three independent GaMD simulations were used for analysis. (Labels: “B” indicate “Bound” low-energy wells of fully active receptors bound to histamine, “I1” indicates “Intermediate” low-energy wells of receptor conformations with smaller and larger TM3–TM6 spacings. “S1”, “S2”, and “S3” illustrate low-energy states with different TM3–TM6 distances, in which histamine was separated from the conserved receptor residue D^3.32^).

Recently, we have developed a dynamic split-luciferase based mini-G protein recruitment assay for the histamine receptor family [52] meeting the requirements of a proximal readout as well as a simple, robust, non-radioactive and homogenous performance [75]. In this assay, HEK293T cells express HR subtypes that are C-terminally fused to the small fragment of the NanoLuc (NlucC) and mini-G proteins N-terminally attached to the large fragment (NlucN). The complementation of the NanoLuc, and thus signal output, is enabled by the recruitment of the mini-G protein to the HR upon receptor activation by a ligand. In the present study, we applied the same concept to investigate the coupling profiles of the H_2_R and H_4_R to engineered G proteins. Therefore, transfectants expressing the H_2_R and H_4_R in combinations with mGs, mGsi, and mGsq were used to probe the functional responses to histamine. We observed that the H_2_R interacted with mGs, mGsi, and mGsq, whereas the H_4_R was selective for binding to mGsi (Figure 1A,B). Notably, histamine was less potent (pEC_50_) and effective (E_max_) in H_2_R-mGsi (pEC_50_ = 5.30 ± 0.06; E_max_ = 26.77 ± 3.47) and H_2_R-mGsq (pEC_50_ = 5.48 ± 0.04; E_max_ = 29.56 ± 3.66) systems than in the H_2_R-mGs system (pEC_50_ = 6.86 ± 0.04; E_max_ = 100%), which represents the canonical interaction pair (Table 1). In other words, we observed a stronger binding of the H_2_R to mGs than to mGsi and mGsq. These observations were consistent with prior findings for the H_2_R of Okashah et al. using BRET-based heterotrimeric G protein sensors and of Inoue et al. using chimeric G proteins [39,44]. In our cell experiments, the H_4_R bound selectively to mGsi yielding a pEC_50_ of 6.60 ± 0.10 and an E_max_ of 100%, (per definition) for the endogenous ligand histamine. Similar results were reported in a previous study using the chimeric G protein approach, in which the H_4_R selectively coupled to members of the Gi family and only slightly to Gα16 of the Gq family [44].

### 2.2. Free Energy Profiles of HR–mG Protein Complexes Were Calculated from GaMD Simulations

**Figure 2:**
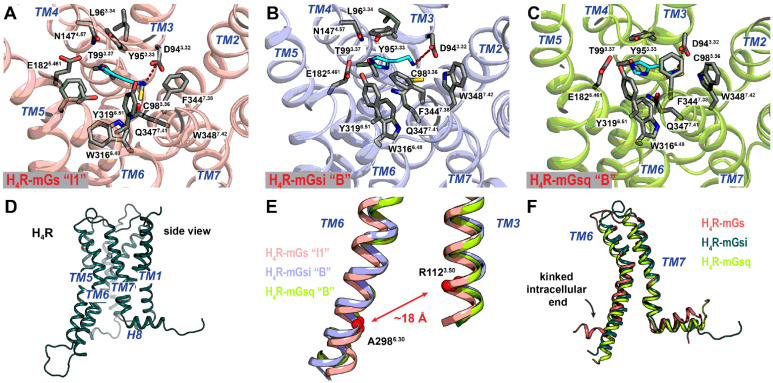
Binding modes of histamine (light blue) within the orthosteric binding pocket of the (**A**) H_4_R–mGs, (**B**) H_4_R–mGsi and (**C**) H_4_R–mGsq complexes. Representative structures of the intermediate “I1” H_4_R–mGs state and of the fully bound “B” H_4_R–mGsi and H_4_R–mGsq states are shown (cf. Figure 1). Contact residues within 4 Å of the ligand are highlighted in sticks (dark gray). The conserved salt bridge between the amino group of histamine and D^3.32^ is highlighted as a red dashed line. (**D**) Side view of the H_4_R (purple). (**E**) TM3–TM6 distances in the in the intermediate state “I1” of H_4_R-mGs (salmon) and the fully bound “B” states of the H_4_R–mGsi (purple) and H_4_R–mGsq (green) complexes. (**F**) Side view of TM6 and TM7 of representative H_4_R–mGs (pink), the H_4_R–mGsi (dark green), and H_4_R–mGsq (green) structures.

In the absence of experimental structures, homology models were built in this study to generate initial complexes of the H_2_R and H_4_R with the mGs, mGsi, and mGsq proteins. A model recently published by Conrad et al. [76] and a model provided by GPCRdb (www.gpcrdb.prg, accessed on 10 August 2021 [77]) were used as simulation starting structures for the H_2_R and the H_4_R, respectively. A computational model of GaMD simulation systems is provided in the Supporting Material (Figure S1). For each of the six systems, we performed three independent 1000 ns GaMD simulations (Table 2). Overall, the H_2_R and H_4_R remained in complex with the ligand histamine and their respective mini-G proteins during the simulations. Considered individually, the same regions were identified as structurally flexible in the complexes. With the exception of the respective mG–α5 helix, which was located in the HR-binding surface, the mini-G proteins mGs, mGsi, and mGsq were structurally more flexible than the membrane-bound H_2_R and H_4_R (see Supporting Material, Figure S2). For the receptors, the largest flexible regions were located at the N- and C-terminal ends and the extracellular loop (ECL) 2 (see Supporting Material, Figure S2A–C). For the H_4_R, a higher flexibility of the intracellular loop (ICL) 3, ECL2, and ECL3 was additionally observed (compare Supporting Material, Figure S2D–F). In all H_2_R and H_4_R complexes, the histamine molecule was located inside the orthosteric binding pocket, but different root-mean-square fluctuations (RMSFs) were observed. Generally, the RMSF of both the proteins and the ligand histamine were the lowest in the systems close to the natural complexes (H_2_R–mGs and H_4_R–mGsi), indicating a favorable complex formation (see Supporting Material, Figure S2). Comparing the H_2_R systems with the H_4_R systems, the replacement of mGs with mGsi and mGsq in the H_2_R systems resulted in a higher structural flexibility of the mG-binding surface (TM5, TM6, H8, and ICLs). In contrast, in H_4_R complexes, the structural variability at the binding surface was comparatively low when mGsi was replaced by mGs or mGsq. Only in H_4_R–mGs were higher fluctuations observed in the intracellular ends of TM5, TM6 (kinked TM6, Figure 2F), ICL2, and ICL3. More characteristic for the H_4_R–mGs and –mGsq complexes was the structural flexibility of the extracellular TM ends, which might be related to the structural flexibility of the histamine ligand. Combined with the cell assay results, we suggest that the more dynamic intracellular surface of H_2_R positively contributes to G protein binding, whereas the increased flexibility of the orthosteric binding pocket in the H_4_R–mGs and –mGsq complexes might be unfavorable for the complex formation.

**Figure 3:**
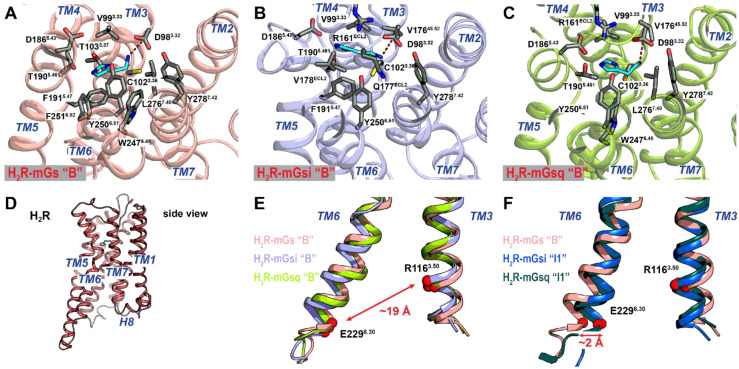
Binding modes of histamine (light blue) within the orthosteric binding pocket of the (**A**) H_2_R–mGs, (**B**) H_2_R–mGsi, and (**C**) H_2_R–mGsq complexes. Structures represent the fully bound state “B” (cf. Figure 1). Contact residues within 4 Å of the ligand are highlighted as sticks (dark gray). The conserved salt bridge between the amino group of histamine and D^3.32^ is given as a red, dashed line. (**D**) Side view of the H_2_R (pink). (**E**) TM3–TM6 distance of the “B” (fully active histamine-bound receptor) states of the H_2_R–mGs (salmon), H_2_R–mGsi (purple), and H_2_R–mGsq (green). (**F**) TM3–TM6 distance of the “B” state of the H_2_R–mGs (salmon) and the “I1” intermediate states of the H_2_R–mGsi (dark green), and H_2_R–mGsq (blue). Residues R^3.50^ and E^6.30^ were used to calculate the TM3–TM6 distance and are given as red spheres.

In the starting structures of the simulations, histamine formed a salt bridge by its charged amino group with the conserved residue D^3.32^ in the orthosteric binding pocket, similarly as postulated in the literature for the entire histamine receptor family [78]. During the simulations, this interaction performed different dynamics in the H_2_R and H_4_R systems. Whereas histamine was permanently bound to D^3.32^ in the H_4_R–mGsi and –mGsq complexes, we were able to sample the disengagement of histamine in the H_2_R complexes and the H_4_R-mGs complex, however, to distinct extent (cf. schematic illustration of the reaction coordinates and detailed time courses in Supporting Material, Figures S3–S5, respectively). The lower abundance of the salt bridge in the H_2_R complexes was in good agreement with the generally lower binding affinity of histamine to H_2_R than to the H_4_R [6]. To investigate whether the presence of the salt bridge was related to the activation state of the receptors, we used the distance between the charge centers of the amino group in histamine and the conserved receptor aspartate D^3.32^ (denoted as D^3.32^–HSM distance) as well as the receptor TM3–TM6 distance (measured between the Cα, C, and N atoms of residues D^3.50^ and E/A^6.30^) as reaction coordinates to calculate the free energy profiles (Figure 1C). The outward movement of TM6 and thus a larger TM3–TM6 spacing is known as one of the key features of GPCR activation and is also suggested to determine G protein selectivity of Gs- and Gi-coupling GPCRs [79,80]. In all systems, the global minima were found for the fully histamine-bound states (D^3.32^–HSM distance of ≈3 Å). In the H_2_R–mGs system, the global minimum was at a TM3–TM6 distance of ≈19 Å (denoted as “B”) (Figure 1C and Figure 2E). In the H_2_R–mGsi and –mGsq complexes, energetic minima at similar TM3–TM6 spacings were present (20 and 19 Å, respectively) (Figure 1C and Figure 2E). However, the global minima of these complexes were located at a slightly smaller TM3–TM6 distance of ~17 Å (denoted as “I1”) (Figure 1C and Figure 2F). The lower capacity of mGsi and mGsq to stabilize the fully active H_2_R conformation was in concordance with the lower E_max_ values (~25–30%) in the mini-G recruitment assay (Table 1). The global minimum of the canonical H_4_R system, H_4_R–mGsi as well as of H_4_R–mGsq were observed at a TM3–TM6 distance of ~18 Å (Figure 1C and Figure 3E). Smaller TM3–TM6 distances in Gi- compared to Gs-coupled GPCRs are related to the smaller α5 helix volume of Gi that requires less three-dimensional space [81]. Interestingly, in the H_4_R–mGs system, the TM3–TM6 distances was even smaller (“I1” state in Figure 2E). Strikingly, this could be attributed to a kinked TM6 helix indicating an unfavorable interaction between the H_4_R and mGs (Figure 2F).

### 2.3. Different Affinities and Binding Conformations of Histamine in H_2_R and H_4_R Complexes

As described, we determined the efficacy (E_max_) and potency (pEC_50_) of histamine at the different complexes in the applied mini-G protein recruitment assay. It is worth noting that complicated relationships might exist between ligand affinity and efficiency [82], and we were interested in whether the experimentally determined pEC_50_ values of histamine would correlate with its binding affinities in the respective HR complexes. Therefore, we calculated the binding energy of histamine in all six complexes using the molecular mechanics generalized Born surface area (MM/GBSA) approach [83,84,85]. Since we observed different dynamics in histamine binding (Figure 1), only frames of the top histamine cluster obtained from the GaMD simulations were used to determine the binding energies. In the H_2_R–mGs complex, histamine was bound ≈1.0 kcal/mol stronger than in the H_2_R–mGsi and H_2_R–mGsq systems (Table 1). This trend was in concordance with the pEC_50_ values of the live cell assay (Table 1). In the H_4_R systems, histamine was most effectively bound to the H_4_R–mGsi complex with a difference of 7.88 kcal/mol or 7.42 kcal/mol compared to the systems containing mGs or mGsq, respectively (Table 1). Although the computational approach gave more pronounced differences in the binding energies of histamine at the H_2_R–mGs and H_4_R–mGsi complexes than the pEC_50_ values obtained in the live cell assay, the calculated binding energies seemed to be consistent with the general dynamics of the systems. In the four complexes with weaker histamine binding (H_2_R–mGs, H_2_R–mGsi, H_2_R–mGsq, and H_4_R–mGs), the disengagement of histamine from D^3.32^ could be detected (Figure 1C). Combined with the lacking interaction in the live cell assay, the more pronounced difference in histamine binding energies might serve as an indicator that the H_4_R is difficult to couple with Gs and Gq.

Recently, the first structure of an HR subtype in an active conformation, the H_1_R, was resolved using cryo-EM, providing insights at the molecular level into the binding mode of the endogenous agonist histamine [60]. The polar interactions with D^3.32^, Y^6.51^, N^5.46^, and T^3.37^ postulated for the entire HR family were confirmed in this structure. Generally, we observed similar binding modes, with only slight differences, for histamine in the representative structures of the “Bound” low-energy states of the H_2_R and H_4_R complexes as in the H_1_R structure. In the H_2_R systems, the orthosteric binding pocket was formed by polar residues D^3.32^, C^3.36^, T^3.37^, D^5.43^, T^5.461^, Y^6.51^, and Y^7.42^ and hydrophobic residues V^3.33^, F^5.47^, F^6.42^, W^6.48^, and L^7.40^ (Figure 3A–C). In all three H_2_R complexes, a salt bridge of the histamine primary amine group with D^3.32^ was formed. The imidazole ring was stabilized by the polar residues D^5.43^ and T^5.461^. In addition, T^3.37^ is further involved in the latter in the H_2_R–mGs system (Figure 3A). Unlike in the H_1_R structure, the sidechain of histamine in the H_2_R–mGs and H_2_R–mGsq complexes is directed toward the hydrophobic residues W^6.48^ and L^7.40^. In the H_2_R–mGsi complex, the histamine sidechain was more likely to be attracted by the backbone of ECL2 residues (V^45.52^, Q177, and V178) (Figure 3B). In the H_4_R systems, the orthosteric binding pocket was surrounded by the polar residues D^3.32^, Y^3.33^, T^3.37^, E^5.461^, Y^5.51^, Q^7.41^, and the hydrophobic residues W^6.48^, F^7.38^, and W^7.42^ (Figure 2A–C). As in the H_2_R complexes, D^3.32^ formed a salt bridge with the primary amine of histamine. The imidazole ring was bound by polar residues Y^3.33^, T^3.37^, and E^5.461^. The structural details of the separated histamine states “S1” and “S2” of the H_2_R complexes and the H_4_R–mGs complex (cf. Figure 1C) were provided in the Supporting Material (Figure S4).

### 2.4. Residue Contacts at the Protein Binding Interface in the H_2_R and H_4_R Systems

**Figure 4:**
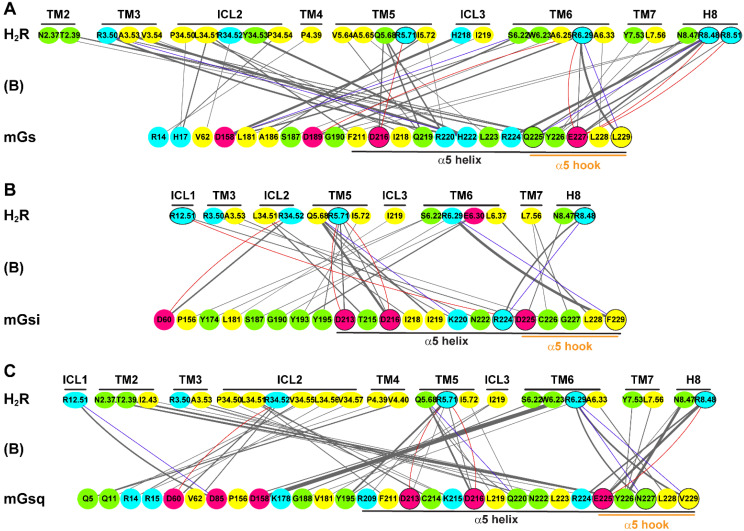
Quantitative residue interactions at the protein interface in the (**A**) H_2_R–mGs, (**B**) H_2_R–mGsi, and (**C**) H_2_R–mGsq (fully active histamine-bound receptor conformations) complexes. The interaction contacts were calculated using the representative low-energy conformation of the systems. Van der Waals, hydrogen bond, and salt bridge interactions are colored in gray, blue, and red, respectively. The line thickness is proportional to the number of contacts of the residue pairs. Hydrophobic, polar, basic, and acidic residues are colored in yellow, green, light blue, and pink, respectively. Important amino acids responsible for polar contacts at the binding interface were highlighted by black circles. For the H_2_R, the BW numbers are used for TM helices and ICL1/2. For ICL3, original index numbers are given. For the mG proteins, the original index numbers are given.

**Figure 5:**
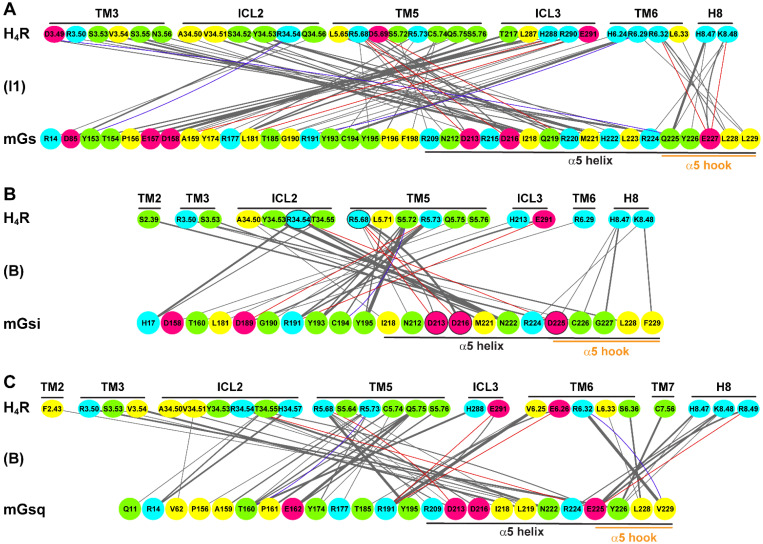
Quantitative residue interactions at the protein interface in the (**A**) H_4_R–mGs, (**B**) H_4_R–mGsi, and (**C**) H_4_R–mGsq (histamine-bound receptor conformations) complexes. The interaction contacts were calculated using the representative low-energy conformation of the systems. Van der Waals, hydrogen bond, and salt bridge interactions are colored in gray, blue, and red, respectively. The line thickness is proportional to the number of contacts of the residue pairs. Hydrophobic, polar, basic, and acidic residues are colored in yellow, green, light blue, and pink, respectively. Important amino acids responsible for polar contacts at the binding interface in the canonical H_4_R–mGsi complex were highlighted by black circles. For the H_4_R, the BW numbers are used for TM helices and ICL1/2. For ICL3, original index numbers are given. For the mini-G proteins, the original index numbers are given.

To examine the coupling profiles of the H_2_R and H_4_R, we extracted residue contact networks for the representative structures of the receptor–mG complexes from GaMD simulations (Figure 4 and Figure 5), noting that the quantitative number of contacts at the protein interfaces is not necessarily decisive due to the different volumes and thus available contact areas of the mG α5 helices. In general, the mGsi α5 helix formed a smaller number of contacts than mGs and mGsq, even in the canonical H_4_R–mGsi system.

The protein contact areas were very similar in the H_2_R complexes but rather different in the H_4_R complexes. The main contacts of the H_2_R with mGs, mGsi, and mGsq involved the receptors ICL2, TM3, TM5, TM6, and H8 (Figure 4). In particular, the negatively charged residues of the mG α5 helices (D213^mG^, D216^mG^, D225^mGsi^/E225^mGsq^, and E227^mGs^) formed salt bridges with similar H_2_R residues leading to comparable mG binding positions. In all H_2_R systems, R^5.71^ (TM5) formed salt bridges with the conserved residues D213^mG^ and D216^mG^ of the α5 helices in the mGs, mGsi, and mGsq (Figure 4). In addition, the α5 hook of the mG proteins was also stabilized by salt bridges in the binding pocket. Accordingly, E227^mGs^ formed three salt bridges with receptor R^6.29^ (TM6), R^8.48^, and R^8.51^ (H8), and E225^mGsq^ formed a single salt bridge with receptor R^8.48^ (Figure 4A,C). In addition to the salt bridge between D225^mGsi^ and R^12.51^ (ICL1), the α5 hook in mGsi was stabilized by hydrogen bonds (R224^mGsi^-R^8.48^ and F229^mGsi^-R^6.29^) in a similar position as in the mGs and mGsq (Figure 4B).

In contrast to the H_2_R systems, the contact surface in the H_4_R complexes were more versatile. In the canonical system, H_4_R–mGsi, a different contact network, was present compared with that in the H_2_R complexes (Figure 5B). For the H_4_R, residues of ICL2 and TM5 contributed the main interactions with the mGsi. Similar to the H_2_R systems, mGsi residues D213^mGsi^ and D216^mGsi^ formed salt bridges with a receptor residue in TM5, R^5.68^. In addition, D225^mGsi^ of the α5 hook formed a salt bridge with R^34.54^ of ICL2.

Unlike in the H_2_R complexes, the α5 hook formed few contacts. Notably, it lacked strong interactions with TM6 and H8. In the H_4_R–mGsq complex, we detected a comparable contact network as in the H_2_R–mGsq complex with the main contacts from ICL2, TM5, TM6, and H8 (Figure 5C). In this system, the conserved mG residue D213^mGsq^ formed a salt bridge with R^5.68^ of the H_4_R. The α5 hook was further closely bound to the H_4_R via the two salt bridges E225^mGsq^–R^34.54^ (ICL2) and E225^mGsq^–R^8.49^ (H8) and by means of a hydrogen bond between V229^mGsq^ and R^6.32^. By contrast, the extremely high number of (hydrophobic) contacts in the H_4_R–mGs complex was particularly striking (Figure 5A). In addition to the few specific interactions of the α5 hook (salt bridge of E227^mGs^ with R^6.32^ (TM6) and K^8.48^ (H8)), these occurred mainly with TM3, ICL2, TM5, and ICL3. Although the H_4_R comprised a larger ICL3 than the H_2_R, the ICL3 was little involved in the protein interface in the canonical system, H_4_R–mGsi. Moreover, after visual analysis of the trajectories, we assumed that mGs stuck to this region due to the large hydrophobic contacts. In combination with the live cell experiments, we therefore suggest an unfavorable interaction for the H_4_R with mGs that would not occur naturally.

### 2.5. α5 Hook Orientation within the Binding Interface of H_2_R and H_4_R Complexes

**Figure 6:**
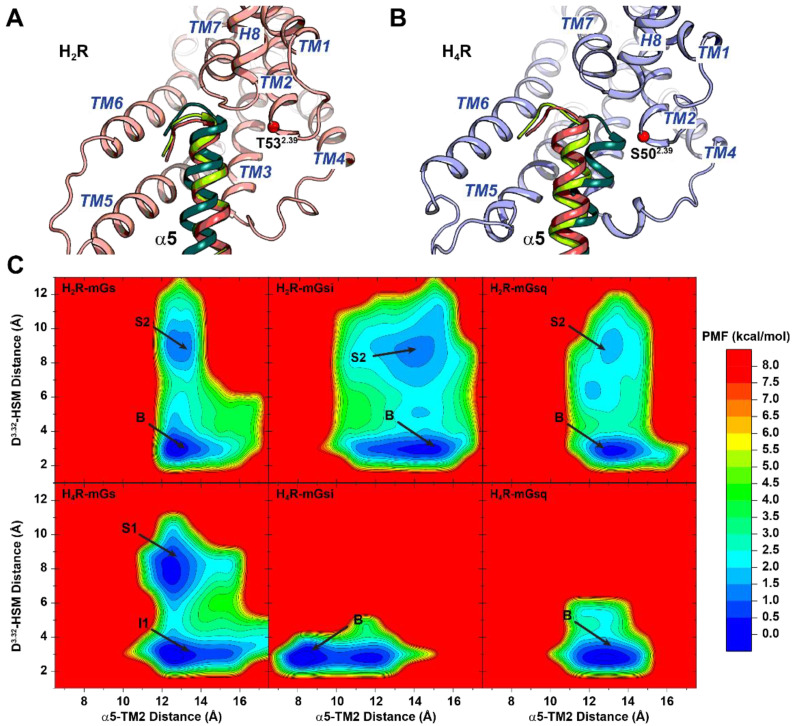
Orientation of the α5 helices of mGs, mGsi, and mGsq in the (**A**) H_2_R complexes and (**B**) H_4_R complexes. The last five residues of α5 helix and residue T/S^2.39^ were used to determine the α5–TM2 distance in the H_2_R and H_4_R complexes, respectively. T/S^2.39^ is shown as red spheres. The H_2_R is depicted in salmon, the H_4_R is depicted in purple, mGs is depicted in pink, mGsi in is depicted dark green, and mGsq is depicted in green, respectively. (**C**) Free energy profiles of GaMD simulations with complexes of either the H_2_R or the H_4_R in combination with mGs, mGsi, or mGsq. Distances of D^3.32^ (CG atom) and the amino group of histamine (Nα atom) as well as of the α5 helix and TM2 were used as reaction coordinates. The α5–TM2 distances were calculated using the geometric center of Cα, C, and N atoms of the last five residues of the mG α5 helix and T/S^2.39^. For each system, three independent GaMD simulations were used for analysis. (Labels: “B” indicates representative low-energy wells of fully active receptors bound to histamine, “I1” indicates low-energy wells of intermediate receptor conformation bound to histamine. “S1” and “S2” indicate low-energy wells containing conformations with histamine separated from D^3.32^, cf. Figure 1).

To further characterize the mG binding in the H_2_R and H_4_R complexes, specific α5 hook positions within the receptor binding cavity were analyzed by the α5–NPxxY distance (cf. Supporting Material Figure S7) and the sideward orientation of the α5 helix (Figure 6). In the literature, it has been described that the penetration depth of G protein α5 helices is characteristic for the G protein families, which is often described by the distance of the α5 to the conserved NPxxY motif (N^7.49^, P^7.50^, Y^7.53^). Commonly, larger outward movements of TM6 in Gs- and Gq-coupled GPCRs move the α5 helix further away from TM7 and thus NPxxY [81]. Accordingly, the mGsi protein was located ~1–2 Å closer to the H_2_R and H_4_R–NPxxY motifs than mGs and mGsq (cf. Supporting Material, Figure S7).

More decisively, we detected differences in the sideward orientation of the α5 helices in the H_2_R and H_4_R systems. In the H_2_R systems, the last five residues of the α5 helices (hook) of mGs, mGsi, and mGsq were in equal position, which we identified as a Gs-like position considering the available GPCR–Gs complexes as the reference (cf. Supporting Material, Figure S8A,D). Notably, only the α5 hook of mGsi adopted a Gi-like orientation in the H_4_R systems (cf. Supporting Material, Figure S8B,E,F). In all systems, we identified a single low-energy well from the free energy profiles calculated using the D^3.32^–histamine distance and α5–TM2 distance as reaction coordinates (Figure 6). The α5 helix in all H_2_R systems as well as inH_4_R–mGs and H_4_R–mGsq systems was oriented toward TM6, giving α5–TM2 distances in the range of ~14 Å. In the H_4_R–mGsi complex, the global minimum was located at an essentially lower α5–TM2 distance of ~8 Å. Thus, the simulation findings suggested that the ability of the H_2_R or rather the inability of the H_4_R to direct the α5 helix to a precise Gs- or Gi-like position contributed to the promiscuous coupling of the H_2_R to Gs, Gi, and Gq and the selective H_4_R binding to Gi.

## 3. Materials and Methods

### 3.1. Generation of Transfectants

The day prior to the transfection, HEK293T were seeded into a 6-well cell culture plate (Sarstedt, Nürmbrecht, Germany) at a density of 0.3 × 10^6^ cells/mL. The cells were transiently transfected with a total amount of 3 µg plasmid DNA using linear polyethyleneimine (PEI, 1 mg/mL in PBS, transfection ratio 1:5 (3 µg DNA + 15 µL PEI)). Therefore, combinations of the following plasmids, the construction of which has been described previously [52], were used: pcDNA3.1 H_2_R–NlucC, pcDNA3.1 H_4_R–NlucC, pIRESpuro3 NlucN–mGs, pIRESpuro3 NlucN–mGsi, and pIRESpuro3 NlucN–mGsq. The cells were incubated for 48 h to allow for an adequate protein expression.

### 3.2. Mini-G Protein Recruitment Assay

The assay protocol has been essentially described before [52,86,87] and was applied with minor modifications as follows. The day prior to the experiment, the cells were detached by trypsin treatment (0.05% trypsin, 0.02% EDTA in PBS), centrifuged at 700× *g* for 5 min, and subsequently resuspended in Leibovitz’s L-15 (L-15) cell culture medium supplemented with 10 mM HEPES (Serva, Heidelberg, Germany). Then, 80 µL of the transfected as well as of the wild-type HEK293T cells (control) were seeded to a white flat-bottom 96-well plate (Brand GmbH + CoKG, Wertheim, Germany) and incubated overnight at 37 °C in a water-saturated atmosphere. On the day of the experiment, the substrate furimazine (Promega, Mannheim, Germany) was 1:100 diluted in L-15 shortly before the experiment, and 10 µL were added to each well. After measuring the baseline luminescence for 15 min at 37 °C using an EnSpire plate reader (Perkin Elmer Inc., Rodgau, Germany), 10 µL of histamine serial dilutions were added to the cells. Finally, luminescent traces were recorded for a further 45 min. Data were analyzed using GraphPad Prism9 software (San Diego, CA, USA). The relative luminescence units (RLU) were corrected for the baseline drift caused by autooxidation of the substrate by dividing all data by the mean luminescence intensity of the HEK293T wild-type control. Thereafter, the baseline luminescence of the respective L-15 control was subtracted. The area under the curve (AUC) of each concentration was normalized to the AUC of 100 µM histamine (100%) obtained in the canonical system (H_2_R-mGs or H_4_R-mGsi, respectively) and L-15 (0%). The logarithmic histamine concentrations were fitted against the normalized AUCs with variable slope (log(c) vs. response—variable slope (four parameters) yielding pEC_50_ and E_max_ values.

### 3.3. Preparation of the H_2_R and H_4_R Complexes

To obtain the H_2_R complexes, the H_2_R–Gs structure that has been published by Conrad et al. was used as template comprising H_2_R residues 15–304 [76]. To obtain the mini-G protein structures mGs, mGsi, and mGsq (according to mGs393, mGsi43, and mGsq71, [46]), homology modeling was performed on the Gs structure using Modeller 9.16 [88]. To avoid artificial charges at the termini of the H_2_R, N-terminal acetyl and C-terminal N-methyl capping groups were added to the structure using Sybyl7.3 (Tripos International, St. Louis, MO, USA (2006) Sybyl 7.3). Coordinates of the ligand histamine were extracted from the template structure and added to the H_2_R–mG complexes. The setup of the complexes for subsequent Gaussian accelerated MD simulations was performed as described previously [89]. During the setup, ff99SB force field parameters [90] were assigned to the complexes, missing hydrogen atoms were added, and the H_2_R disulfide bond C91–C174 was created using tleap. The energy minimization and equilibration steps were essentially performed as described before [89] using Amber17 [91] and Gromacs 2016.5 [92]. In order to embed the H_2_R into a membrane, the structure was overlaid with a pre-equilibrated dioleylphosphatidylcholine (DOPC) bilayer (gaff force field, [93]) and solvated with SPC water [94]. Therefore, the pseudo-atom entries in the 3SN6 OPM entry containing the position of extracellular and intracellular membrane layer have been used.

The H_4_R systems were obtained using the homology model of the receptor provided by GPCRdb (www.gpcrdb.org (accessed on 10 August 2021), Version: 3 September 2019, mainly based on M_2_R structure; pdb-id: 4MQT) [77] as template. The utilized model comprised residues 6–380. Of note, the ICL3 was truncated (69 total missing amino acids) and only consisted of the first 8 (-CQSHPGLT-) and last 5 residues (-LHQRE-) instead of the native, 82 amino acid-sized ICL3. Amber coordinates and topology files were generated for the downloaded pdb file using *ambpdb*, and the disulfide bond C87–C164 was created using *tleap*. Initially, the structure was solvated in a TIP3 waterbox with Cl^−^ as counter ions and minimized as performed for the H_2_R structures. To set up the H_4_R–mG complexes, a loop refinement of the ICL3 has been performed using the ModLoop web server (www.modbase.compbio.ucsf.edu/modloop, accessed on 10 August 2021 [95,96] to avoid clashes between the receptor and mG protein structures. The refined H_4_R structure and the mG homology models that have been generated in complex with the H_2_R were aligned to the M_2_R structure in complex with Gi (pdb-id.: 6OIK) to extract appropriate coordinates. Thereafter, similar protocols [89] as for the H_2_R–mG complexes were performed to prepare the H_4_R systems (H_4_R-mGs, H_4_R-mGsi and H_4_R–mGsq) bound to the ligand histamine. A computational model of GaMD simulation systems and a sequence alignment of the chimeric mini-G proteins and the “parental” G protein subunit Gαs are provided in the Supporting Material (Figures S1 and S9, respectively).

### 3.4. Gaussian Accelerated Molecular Dynamics (GaMD) Simulations

In GaMD, a harmonic, Gaussian-distributed boost potential is applied to biomolecules to smooth the potential energy surface and reduce energy barriers [67]. When the system potential $V\left(\vec{r}\right)$ is lower than a reference energy *E*, the modified potential $V^{*}\left(\vec{r}\right)$ is calculated as:

(1) $$V^{*}\left(\vec{r}\right)=V\left(\vec{r}\right)+∆V\left(\vec{r}\right)\;∆V\left(\vec{r}\right)=\{\begin{array}{c} \frac{1}{2}k{\left(E-V\left(\vec{r}\right)\right)}^{2},\;V\left(\vec{r}\right)<E\;\\ \;0,\;V\left(\vec{r}\right)\geq E, \end{array}$$

where *k* is the harmonic force constant. The two adjustable parameters *E* and *k* are automatically determined on three enhanced sampling principles as described before [67]. In summary, *E* needs to be in the range:

(2) $$V_{max}\leq E\leq V_{min}+\frac{1}{k},$$

where $V_{min}$ and $V_{max}$ are the minimum and maximum potential energies of the system. To ensure that Equation (2) is valid, *k* is defined as $k=k_{0}\cdot 1/\left(V_{max}-V_{min}\right)$ and thus $0<k_{0}\leq 1$. To enable an accurate energetic reweighting using cumulant expansion to the second order, the third standard deviation of ∆V needs to be small enough: $\sigma_{∆V}=k\left(E-V_{avg}\right)\sigma_{V}\leq \sigma_{0}$, where $V_{avg}$ and $\sigma_{V}$ are the average and standard deviation of the system potential energies and $\sigma_{∆V}$ is the standard deviation of ∆V with $\sigma_{0}$ as a user-specified upper limit for accurate reweighting. When *E* is set to the lower bound $E=V_{max}$, according to Equation (2), $k_{0}$ can be calculated as:

(3) $$k_{0}=\min\left(1.0,k_{0}^{\prime }\right)=min\left(1.0,\frac{\sigma_{0}}{\sigma_{V}}\cdot \frac{V_{max}-V_{min}}{V_{max}-V_{avg}}\right),$$

Alternatively, when the threshold energy *E* is set to its upper bound $E=V_{min}+1/k$, $k_{0}$ is set to:

(4) $$k_{0}=k_{0}^{\prime \prime }\equiv \left(1-\frac{\sigma_{0}}{\sigma_{V}}\;\right)\cdot \frac{V_{max}-V_{min}}{V_{avg}-V_{min}},$$

if $k_{0}^{\prime \prime }$ is found between 0 and 1. Otherwise, $k_{0}$ is calculated using Equation (3).

### 3.5. Energetic Reweighting of GaMD Simulations

To calculate the potential of the mean force (PMF) and energetically reweight GaMD simulations, the probability distribution along a reaction coordinate is written as $p^{*}\left(A\right)$. Given the boost potential ∆V(r) of each frame, $p^{*}\left(A\right)$ can be reweighted to recover the canonical ensemble distribution p(A), as:

(5) $$p\left(A_{j}\right)=p^{*}\left(A_{j}\right)\frac{\langle e^{\beta ∆V\left(r\right)}\rangle_{j}}{\sum_{i=1}^{M}\langle p^{*}\left(A_{i}\right)e^{\beta ∆V\left(r\right)}\rangle_{i}},\;j=1,\ldots ,\;M,$$

where *M* is the number of bins, $\beta =k_{B}T$, and $\langle e^{\beta ∆V\left(r\right)}\rangle_{j}$ is the ensemble-averaged Boltzmann factor of ∆V(r) for simulation frames found in the *j*th bin. The ensemble-averaged reweighting factor can be approximated using cumulant expansion:

(6) $$\langle e^{\beta ∆V\left(r\right)}\rangle =exp\left\{\sum_{k=1}^{\infty }\frac{\beta^{k}}{k!}C_{k}\right\},\;$$

where the first two cumulants are given by:

(7) $$\begin{array}{c} C_{1}=\langle ∆V\rangle ,\\ C_{2}=\langle ∆V^{2}\rangle -{\langle ∆V\rangle }^{2}=\sigma_{v}^{2} \end{array}$$

The boost potential obtained from GaMD simulations usually follows near-Gaussian distribution [69,70]. Thus, the cumulant expansion to the second order provides a good approximation for computing the reweighting factor [67,97]. The reweighted free energy $F\left(A\right)=-k_{B}T\;\ln\;p\left(A\right)$ is calculated as:

(8) $$F\left(A\right)=F^{*}\left(A\right)-\sum_{k=1}^{2}\frac{\beta^{k}}{k!}C_{k}+F_{c}$$

where $F^{*}\left(A\right)=-k_{B}T\;\ln\;p^{*}\left(A\right)$ is the modified free energy obtained from GaMD simulation and $F_{c}$ is a constant.

### 3.6. Simulation Protocol

GaMD has been implemented in the AMBER software package, so that all molecular dynamics (MD) simulations could be performed using *Amber18* [98]. During the simulations, periodic boundary conditions were given. The *SHAKE* algorithm was applied to remove the bond stretching freedom of all hydrogen-containing bonds. The GaMD simulations were preceded by a short conventional MD (cMD) simulation of 10.4 ns for the statistical collection of boost parameters (*V_max_*, *V_min_*, *V_avg_*, and *σ_V_*), which was followed by 32 ns “dual-boosted” MD simulations using the calculated parameters (2 fs time steps), in which boost parameters were imposed on the total potential energy and the dihedral energy terms. The reference energy was set to the lower bound (*E = V_max_*), and the boost parameters were updated every 400,000 steps (800 ps). The upper limit of the standard deviation of both the boost potential, the total potential energy, and the dihedral energy were defined as *σ*_OP_ = *σ*_OD_ = 6.0 kcal/mol. After the preparatory stage, 3 × 1000 ns of GaMD production runs were performed for the H_2_R and H_4_R systems. Table 2 gives an overview of the average boost potentials that have been applied for the different systems and simulation runs.

### 3.7. Structural Analysis

For subsequent analysis, periodic boundaries were removed from the trajectories, and the molecules were centered to the receptors’ transmembrane domains using cpptraj of Amber18 software (San Franscicso, CA, USA) [98]. Furthermore, cpptraj was used for the structural analysis of all distances and angles. Contacts between the H_2_R/H_4_R and mini-G proteins were assessed using the nativecontacts command with a specified distance of 5 Å. Binding energies of the ligand histamine were determined using the mm_pbsa.pl script using IGB = 2 and further parameters in default option according to the MM/GBSA method [83,84,85]. Plots were created using GraphPad Prism9 (GraphPad Software, San Diego, CA, USA), Cytoscape (Shannon P., 2003), and Origin2021 (OriginLab Corporation, Northampton, MA, USA), and the structural visualization was performed using pymol (The PyMOL Molecular Graphics System, Version 2.0 Schrödinger, LLC, New York, NY, USA).

Important reaction coordinates were identified from the simulation trajectories such that they involved system dynamic regions and could be used to differentiate conformational states of the HR–mG protein complexes. The observed dynamic regions included the agonists, the receptor TM6 helix, and the C terminus of the mG α5 helix. Therefore, the distance between the charged amine group of histamine and the highly conserved D^3.32^ and the receptor TM3 and TM6 intracellular ends were selected as reaction coordinates. The distance between the conserved NPxxY motif in the TM7 intracellular end of the receptors and the C terminus of the mG α5 helix (α5 hook) was used to characterize the HR–mG protein interactions. Furthermore, the TM2–α5 distance was calculated to estimate the α5 hook orientation inside the binding cavity. Particularly, distances were calculated between the backbone (Cα, C, and N) atoms using the center of mass of the following residues. For the TM3–TM6 distance, residues R^3.50^ and E/A^6.30^, and for the NPxxY–α5 distance, residues N^7.49^, P^7.50^, and Y^7.53^ as well as the last five residues of the mG α5 helix were used. To calculate the α5–TM2 distance, the last five residues of the mG α5 helix and T/S^2.39^ were used. The time courses of these reaction coordinates obtained from the GaMD simulation were plotted in the Supporting Material (Figures S4 and S5). Root-mean-square fluctuations (RMSFs) were calculated for the protein residues and histamine, averaged over three independent GaMD simulations, and color coded for the schematic representation of each complex system (cf. Supporting Material, Figure S2). The representative low-energy conformations of the HR–mG protein complexes were used to compute their residue contact network at the binding interface of the proteins using the nativecontacts command of cpptraj with a distance cutoff of 4 Å. For two-dimensional visualization, the software Cytoscape [99] was utilized to plot the residue contact network.

To recover the original free energy or potential of mean force (PMF) profiles of the four HR–G protein complex systems, GaMD simulations were reweighted using the PyReweighting toolkit [97]. PMF profiles were computed using the combined trajectories from all three independent GaMD simulations (3 × 1000 ns length) for each system. A bin size of 1.0 Å was used, and the cutoff was set to 500 frames for 2D PMF calculations. The 2D PMF profiles were obtained for each simulation system regarding D^3.32^–histamine distance in combination with the TM3–TM6 distance, the NPxxY–α5 distance, and the TM2–α5 distance.

## 4. Conclusions

In this study, we have investigated the H_2_R and H_4_R in complex with three types of mini-G proteins, mGs, mGsi, and mGsq, respectively. The results obtained in cellular experiments and GaMD simulations were in good agreement providing important insights into the mechanism of engineered G protein coupling to both receptors. In the mini-G protein recruitment assay using HEK293T cells transiently expressing combinations of either the H_2_R or the H_4_R and mGs, mGsi, and mGsq, we observed a promiscuous coupling of the H_2_R to mGs, mGsi, and mGsq upon activation by histamine. Regardless, the H_2_R preferred the mGs binding over mGsi or mGsq binding revealed by pEC_50_ and E_max_ values (Figure 1A,B; Table 1). In contrast, the H_4_R selectively bound to mGsi. In GaMD simulations, a similar preference was observed due to the lower structural flexibility of the H_2_R–mGs and H_4_R–mGsi complexes. In the simulations, a salt bridge between Nα of the endogenous ligand histamine and D^3.32^ was present, which is conserved among histamine receptor subtypes [60,78]. Nevertheless, the binding energies of histamine were different in the complexes. The binding energy determined by MM/GBSA was stronger in the H_4_R than in H_2_R complexes, which was in concordance with a reported higher binding affinity of histamine to the H_4_R [100,101]. Another important aspect was that the H_2_R systems were more dynamic than the H_4_R complexes, leading to larger fluctuations in the TM3–TM6 distances in the H_2_R–mGsi and H_2_R–mGsq being considered as common indicators for GPCR activation [79,102]. In agreement with the higher pEC_50_ and less E_max_ values, the mGsi and mGsq proteins were less able to stabilize the fully active H_2_R conformation (TM3–TM6 distance of ~19–20 Å), and the systems also visited conformations with a smaller TM3–TM6 spacing (Figure 1C). As observed for many Gi-coupling GPCRs, the TM3–TM6 distance in the fully active H_4_R in the H_4_R–mGsi complex was lower (~18 Å) than in the canonically Gs-coupled H_2_R. Strikingly, the overall TM3–TM6 distances in the H_4_R–mGs complex (“I1” state) were different from the H_4_R–mGsi (“B” state).

The detailed inspection of the protein interfaces revealed similar residue contributions and binding orientations of mGs, mGsi, and mGsq in the H_2_R binding cavity. The α5 helices were in Gs-like orientation toward TM6 that has been stabilized by specific α5 hook polar interactions with TM6 and H8. Contrarily, the contact network in the H_4_R complexes was more versatile. Notably, only the mGsi α5 helix was in Gi-like orientation toward TM2, which was potentially enabled by the specific α5 helix interactions with TM5 and ICL2 and fewer contacts of the α5 hook. In the H_4_R–mGs and –mGsq complexes, mGs and mGsq were in a similar position as in the H_2_R complexes. Moreover, excessively strong hydrophobic mGs interactions with TM3, ICL3, and TM5 led to the assumption of an unfavorable interaction.

In summary, a coherent overall picture of the H_2_R and H_4_R coupling profiles to engineered G proteins has emerged by the combination of biochemical as well as computational experiments supporting the following: The H_2_R can bind promiscuously to Gs, Gi, and Gq, whereas the H_4_R binds selectively to Gi. Our results suggested that particularly the structural flexibility as well as the ability of a receptor to locate the α5 hook in a precise position, e.g., Gs-like or Gi-like position (Figure 6), might contribute to the coupling behavior of GPCRs. However, these results may not be generalized to the entire G protein classes but rather provide an indication of the preferred Gα subunit. Mini-G proteins are chimeric G proteins derived from Gαs, Gαi_1_, and Gαq proteins. Thus, it remains unclear whether or in which way H_2_R and H_4_R would form complexes with Gi family members Gαi_3_ and Gαo or to Gα14 and Gα15 of the Gq family having diverse α5 helices within the Gα classes. In particular, future studies are required focusing on the H_4_R interactions with the Gq family subtype Gα15, which has been demonstrated by Inoue et al. [44], to explain Ca^2+^ signals that have been obtained earlier using cells co-expressing the H_4_R and Gα15 [30] but were missing in HEK cells recombinantly expressing the H_4_R alone [27]. Furthermore, experiments comprising holistic cellular responses [103] or the investigation of G protein-dependent second messengers along the signal cascade will be advantageous to fully unravel H_2_R and H_4_R signaling.

## Figures and Tables

**Table 1:** Comparison of biochemical and computational ligand binding data. Potencies (pEC_50_) and efficacies (E_max_) of histamine obtained in the split-Nanoluciferase-based mini-G protein recruitment assay using HEK293T cells transiently expressing either the H_2_R or the H_4_R in combination with mGs, mGsi, or mGsq. Presented data are of three independent experiments (*N* = 3) each performed in triplicate. Binding energies (kcal/mol) of histamine obtained in the same complexes applying the MM/GBSA method to the GaMD trajectories. For each system, 60,000 frames of the top histamine cluster were analyzed. Averages ± SD are given.

	Mini-G Protein Recruitment Assay		MM/GBSA
	pEC_50_ ± SEM	E_max_ ± SEM (%)	Binding Energy ± SD (kcal/mol)
H_2_R–mGs	6.86 ± 0.04	100	−17.23 ± 3.44
H_2_R–mGsi	5.30 ± 0.06	26.77 ± 3.47	−16.57 ± 3.81
H_2_R–mGsq	5.48 ± 0.04	29.56 ± 3.66	−16.25 ± 6.35
H_4_R–mGs	n.d.	−13.51 ± 5.50	−20.03 ± 5.44
H_4_R–mGsi	6.60 ± 0.10	100	−27.91 ± 4.69
H_4_R–mGsq	n.d.	13.82 ± 0.28	−20.49 ± 6.78

**Table 2:** Summary of the boost potentials applied in the Gaussian accelerated molecular dynamics (GaMD) simulations. In all GaMD simulations, the total potential energy (E_pot_) and the dihedral energy (E_dihedral_) were boosted (“dual-boost”). Average ± SD of the corresponding boost potentials (∆V_pot_ and ∆V_dihedral_) are given for the different simulations of the H_2_R and H_4_R systems.

System	N_atoms_	Simulation	Length (ns)	∆V_pot_ (kcal/mol)	∆V_dihedral_ (kcal/mol)
H_2_R–mGs	128,856	GaMD1	1000	7.53 ± 3.15	6.40 ± 2.65
		GaMD2	1000	7.50 ± 3.15	6.50 ± 2.68
		GaMD3	1000	7.53 ± 3.16	6.36 ± 2.64
H_2_R–mGsi	128,819	GaMD1	1000	7.87 ± 3.23	5.93 ± 2.55
		GaMD2	1000	7.86 ± 3.23	5.88 ± 2.54
		GaMD3	1000	7.85 ± 3.22	5.98 ± 2.56
H_2_R–mGsq	128,864	GaMD1	1000	7.39 ± 3.13	6.32 ± 2.63
		GaMD2	1000	7.36 ± 3.13	6.34 ± 2.64
		GaMD3	1000	7.37 ± 3.13	6.52 ± 2.68
H_4_R–mGs	129,747	GaMD1	1000	7.87 ± 3.23	6.25 ± 2.62
		GaMD2	1000	7.86 ± 3.23	6.08 ± 2.58
		GaMD3	1000	7.84 ± 3.23	6.45 ± 2.66
H_4_R–mGsi	129,737	GaMD1	1000	8.05 ± 3.26	6.03 ± 2.57
		GaMD2	1000	8.02 ± 3.25	6.42 ± 2.65
		GaMD3	1000	8.04 ± 3.26	6.19 ± 2.60
H_4_R–mGsq	129,788	GaMD1	1000	8.09 ± 3.27	6.55 ± 2.68
		GaMD2	1000	8.21 ± 3.29	6.57 ± 2.68
		GaMD3	1000	8.22 ± 3.30	6.43 ± 2.65

## Supplementary Materials

The following are available online at https://www.mdpi.com/article/10.3390/ijms221810047/s1.
