# Supplementary material for: Specific Engineered G Protein Coupling to Histamine Receptors Revealed from Cellular Assay Experiments and Accelerated Molecular Dynamics Simulations
Source: Int J Mol Sci. 2021 Sep 17;22(18):10047. doi: 10.3390/ijms221810047 (PMC8467750; doi:10.3390/ijms221810047)
Supplement: Supplementary file 1 [file ijms-22-10047-s001.zip › ijms-1359344-supplementary.pdf]

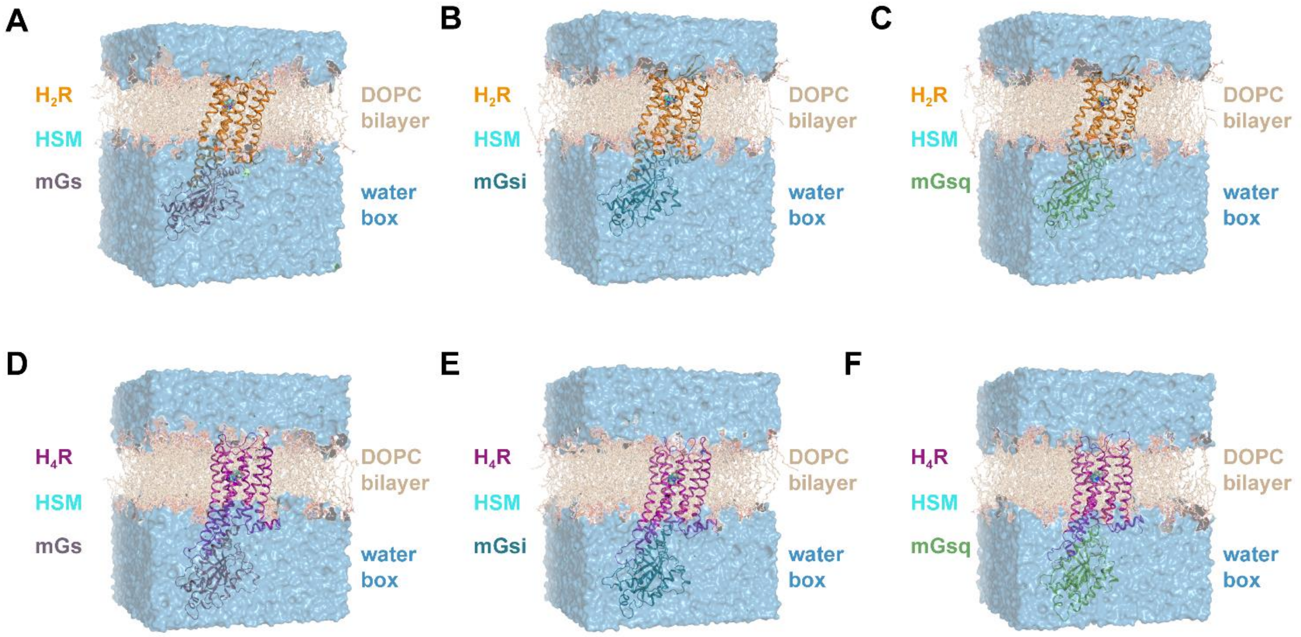

Figure S1. Computational model of GaMD simulation systems containing the H_2R in complex with A) mGs, B) mGsi and C) mGsq as well as the H_4R in complex with D) mGs, E) mGsi and F) mGsq. The histamine (HSM)-bound receptors complexed by mini-G proteins were embedded into a dioleoylphosphatidylcholine (DOPC) bilayer and solvated in a TIP3 water box with Cl- as counter ions.

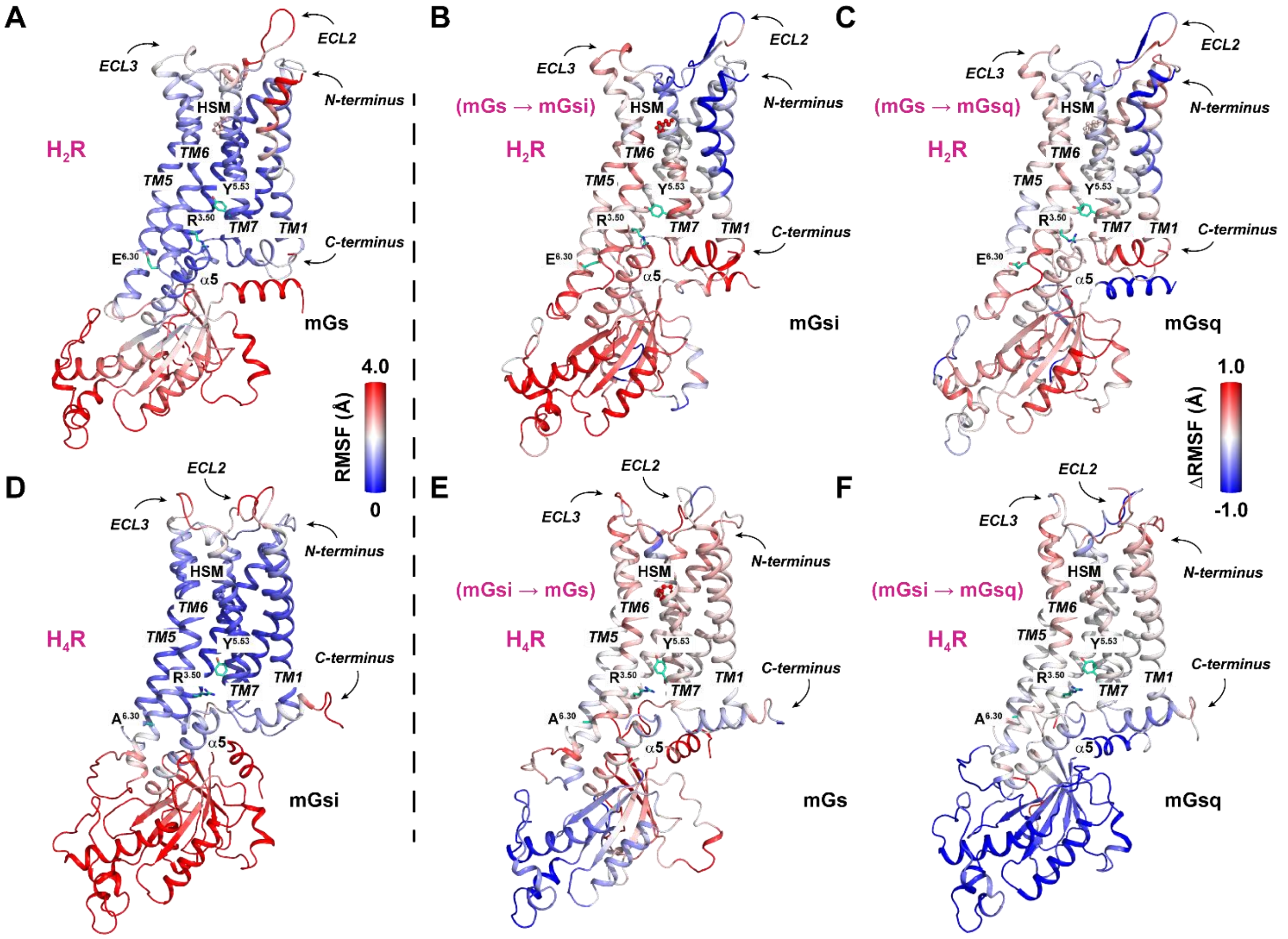

Figure S2. Comparison of the structural flexibility in H2R and H4R complexes with mini-G proteins. A) Overall structural flexibility (RMSF) of the H2R-mGs complex and changes in structural flexibility (ΔRMSF) in the H2R complexes, when mGs was exchanged by mGsi (B) or mGsq (C). D) Overall structural flexibility (RMSF) of the H4R-mGsi complex and changes in structural flexibility (ΔRMSF) in the H4R complexes, when mGsi was exchanged by mGs (E) or mGsq (F). For RMSFs, a color scale of 0.0 Å (blue) to 4.0 Å (red) was used. In case of ΔRMSF, the color scale ranged from -1.0 Å (blue) to 1.0 Å (red). (Δ)RMSF values were assigned to the starting structure of each system.

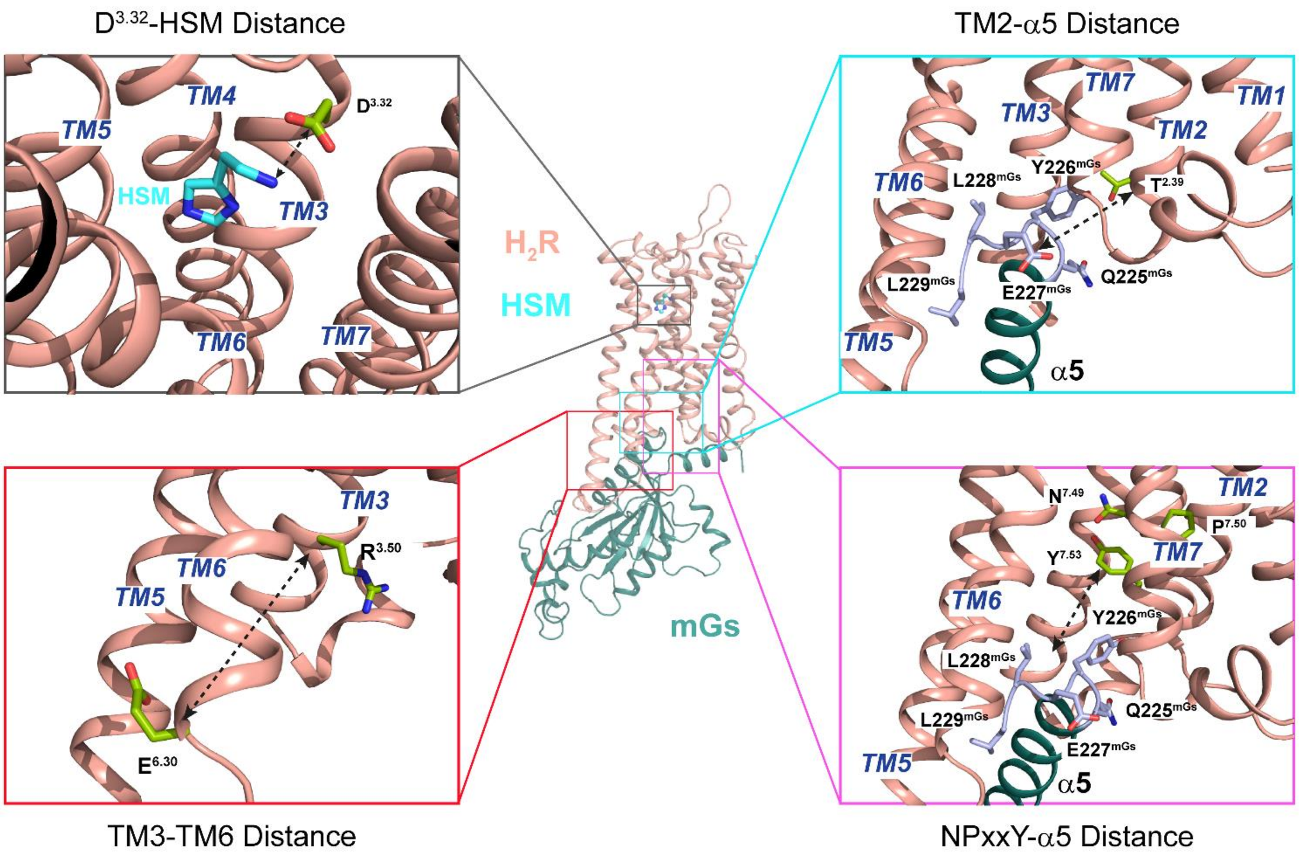

Figure S3. Schematic illustration of the reaction coordinates used for energetic reweighting exemplarily shown for the H_2R in complex with mGs. The distance between $D^{3.32}$ and histamine (HSM) was calculated using the Cγ atom of $D^{3.32}$ and Nα of histamine. The distance between TM3 and TM6 was assessed using the Cα, C and N atoms of residues $R^{3.50}$ and $E^{6.30}$. The distance between the NPxxY motif and α5 helix of mGs was calculated using the center of mass of the NPxxY motif ($N^{7.49}$, $P^{7.50}$ and $Y^{7.53}$) and the last five residues of mGs α5 helix (Q225, Y226, E227, L228 and L229). The distance between TM2 of the H_2R and the α5 helix of mGs was determined using the Cα, C and N atoms of $T^{2.39}$ and the geometric center of the last five residues of mGs α5 helix (Q225, Y226, E227, L228 and L229).

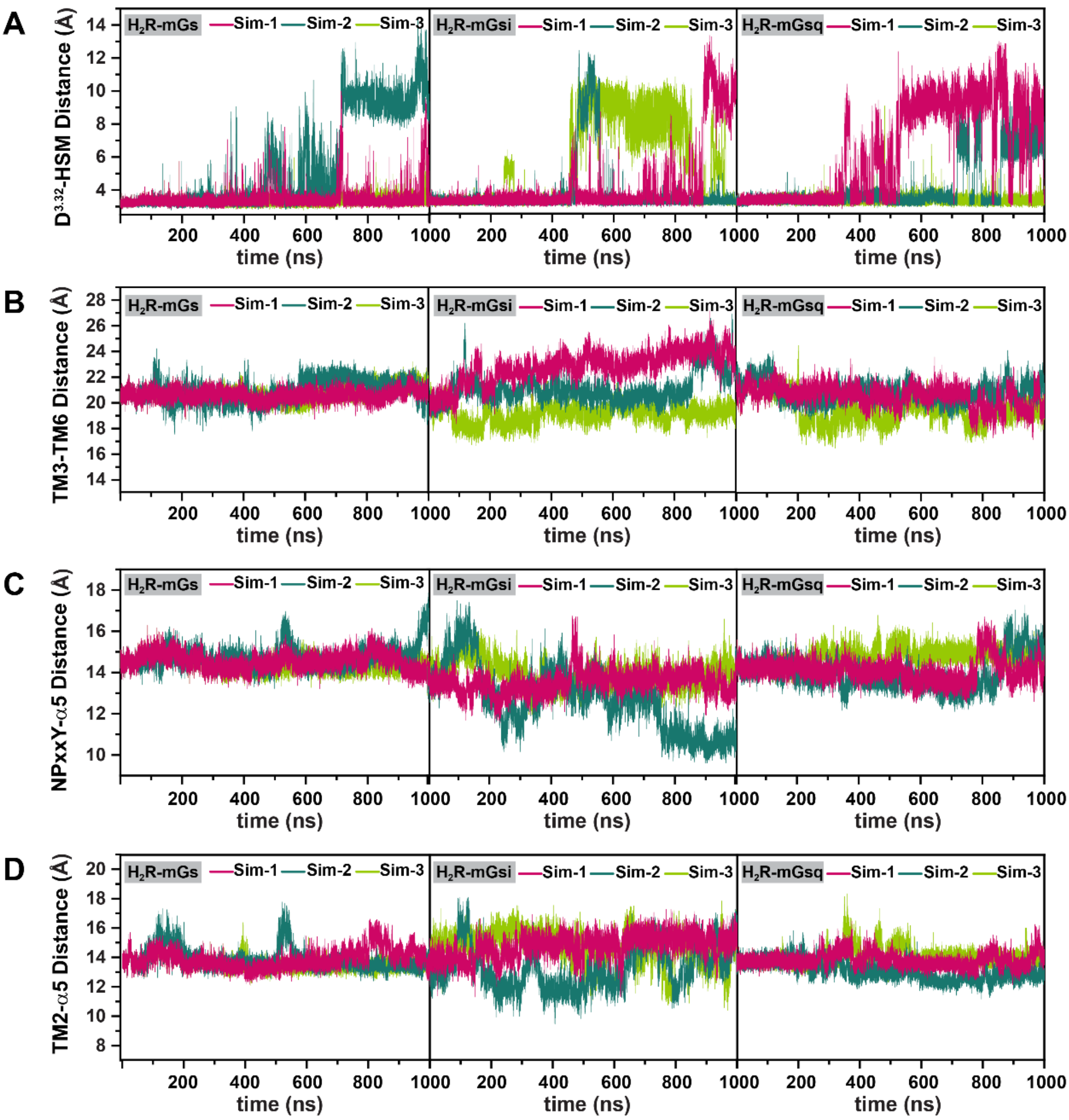

Figure S4. Time courses of the reaction coordinates in H_2R systems (H_2R-mGs, H_2R-mGsi and H_2R-mGsq) used for energetic reweighting. A) Distance between $D^{3.32}$ and histamine (HSM) using the CG atom of $D^{3.32}$ and Nα of histamine. B) Distance between TM3 and TM6 of the H_2R. The Cα, C and N atoms of residues $R^{3.50}$ and $E^{6.30}$ were used to calculate the distance. C) Distance between the NPxxY motif and α5 helix of the respective mini-G protein. The distance was calculated using the center of mass of the NPxxY motif and the last five residues of α5 helix. D) Distance between TM2 of the H_2R and the α5 helix of the respective mini-G protein. To calculate the distance, the Cα, C and N atoms of $T^{2.39}$ and the geometric center of the last five residues of α5 were used.

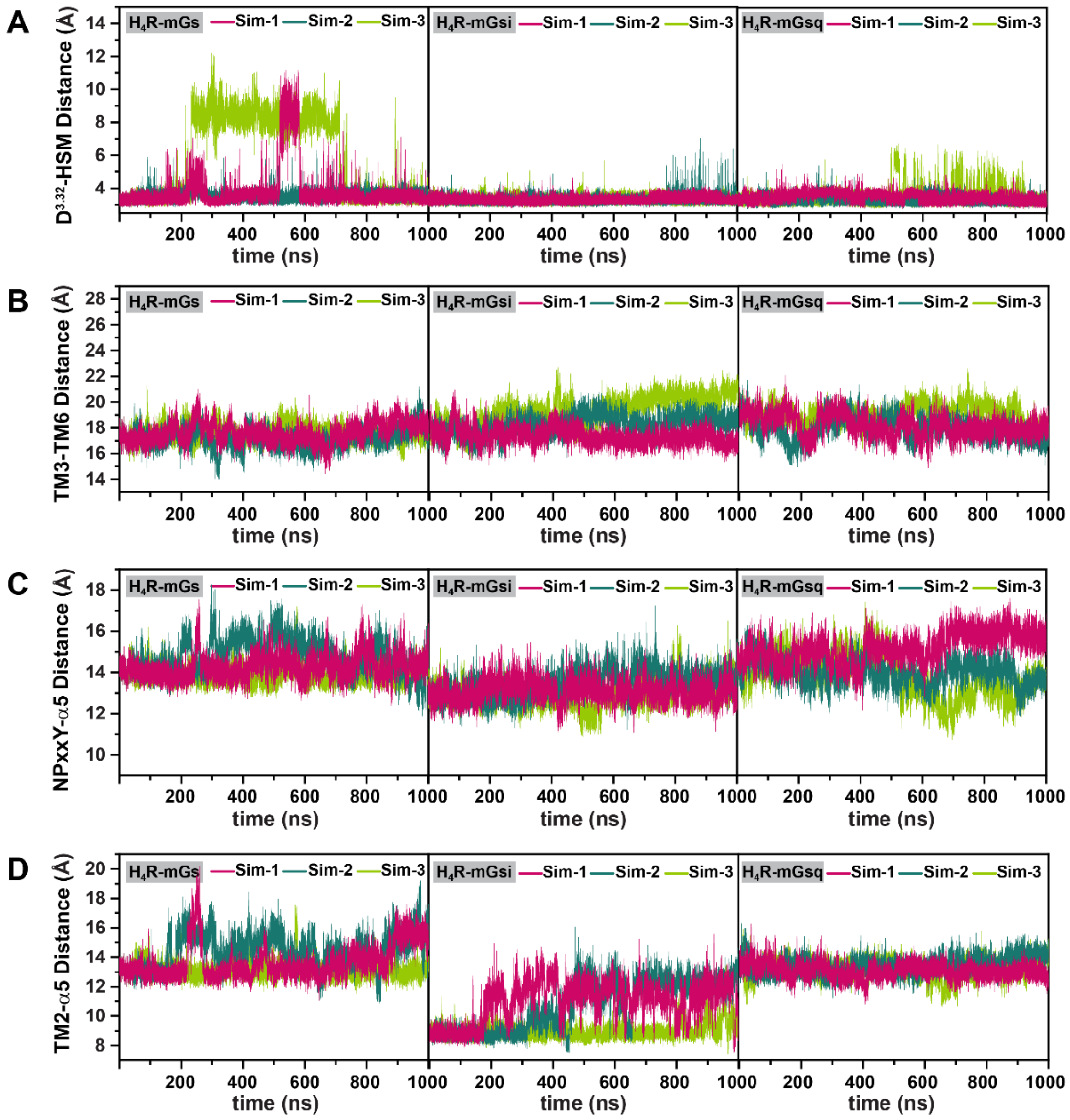

Figure S5. Time courses of the reaction coordinates in H4R systems (H4R-mGs, H4R-mGsi and H4R-mGsq) used for energetic reweighting. A) Distance between $D^{3.32}$ and histamine (HSM) using the CG atom of $D^{3.32}$ and Nα of histamine. B) Distance between TM3 and TM6 of the H4R. The Cα, C and N atoms of residues $R^{3.50}$ and $A^{6.30}$ were used to calculate the distance. C) Distance between the NPxxY motif and α5 helix of the respective mini-G protein. The distance was calculated using the center of mass of the NPxxY motif and the last five residues of α5 helix. D) Distance between TM2 of the H4R and the α5 helix of the respective mini-G protein. To calculate the distance, the Cα, C and N atoms of $S^{2.39}$ and the geometric center of the last five residues of α5 were used.

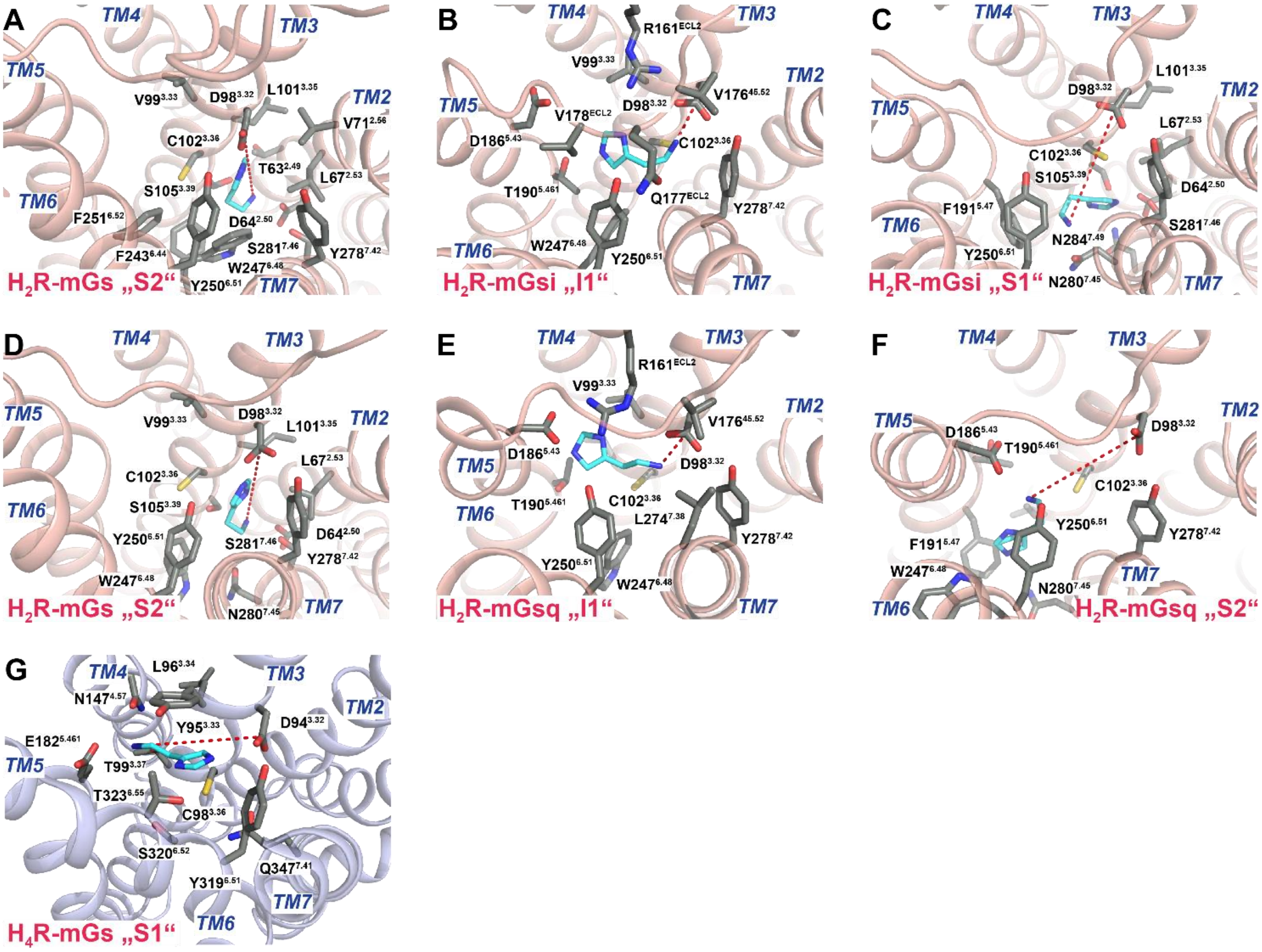

Figure S6. Binding modes of histamine (light blue) within the orthosteric binding pocket of the H$_2$R (salmon) and the H$_4$R (purple). Structures representing the separated histamine state “S2” in H$_2$R-mGs (**A**), the intermediately bound state “I1” (**B**) and the separated histamine states “S1” (**C**) and “S2” (**D**) in H$_2$R-mGsi, the intermediately bound state “I1” (**E**) and the separated histamine states “S2” (**F**) in H$_2$R-mGsq complexes, as well as the separated histamine state “S1” in the H$_4$R-mGs complex (**G**) are shown. Contact residues within 4 Å of the ligand are highlighted as sticks (dark grey). The histamine-D$^{3.32}$ distance is highlighted with a red, dashed line.

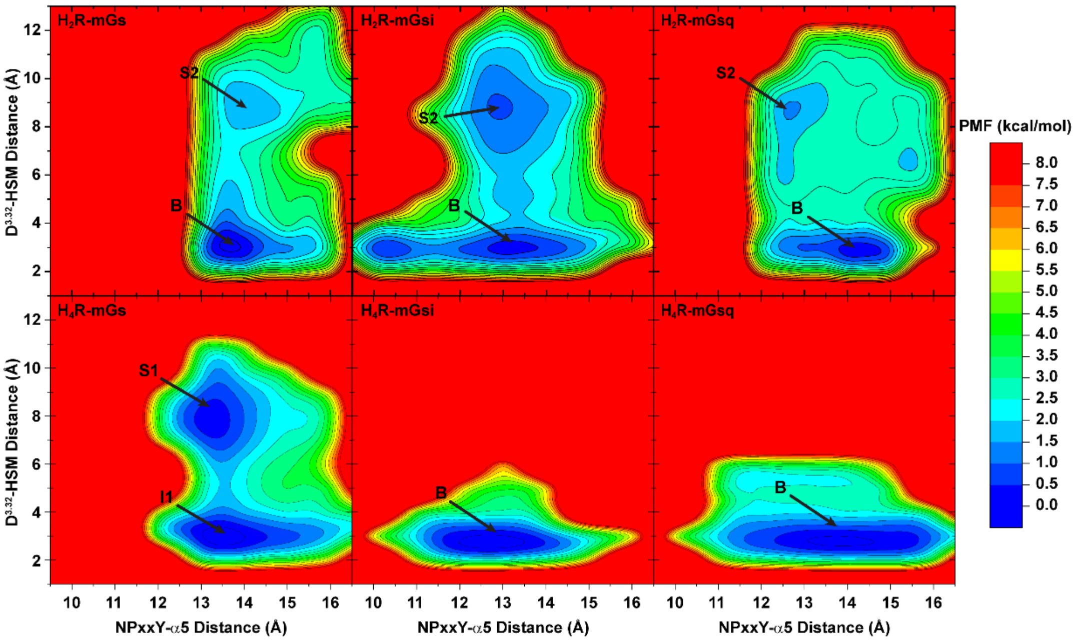

Figure S7. Free energy profiles of GaMD simulations with complexes of either the H_2R or the H_4R in combination with mGs, mGsi or mGsq. Distances (Å) between $D^{3.32}$ (CG atom) and the amino group of histamine (Nα atom) as well as of the NPxxY - α5 helix distance were used as reaction coordinates. The NPxxY distance was determined using the center-of-mass (COM) distance between the receptors' NPxxY motif and the last 5 residues of the mG α5 helix. For each system, three independent GaMD simulations were used for analysis. (Labels: "B" indicates representative low energy wells of fully active receptors bound to histamine, "I1" indicates low energy wells of intermediate receptor conformation bound to histamine. "S1" and "S2" indicate low energy wells containing conformations with histamine separated from $D^{3.32}$, cf. Figure 1).

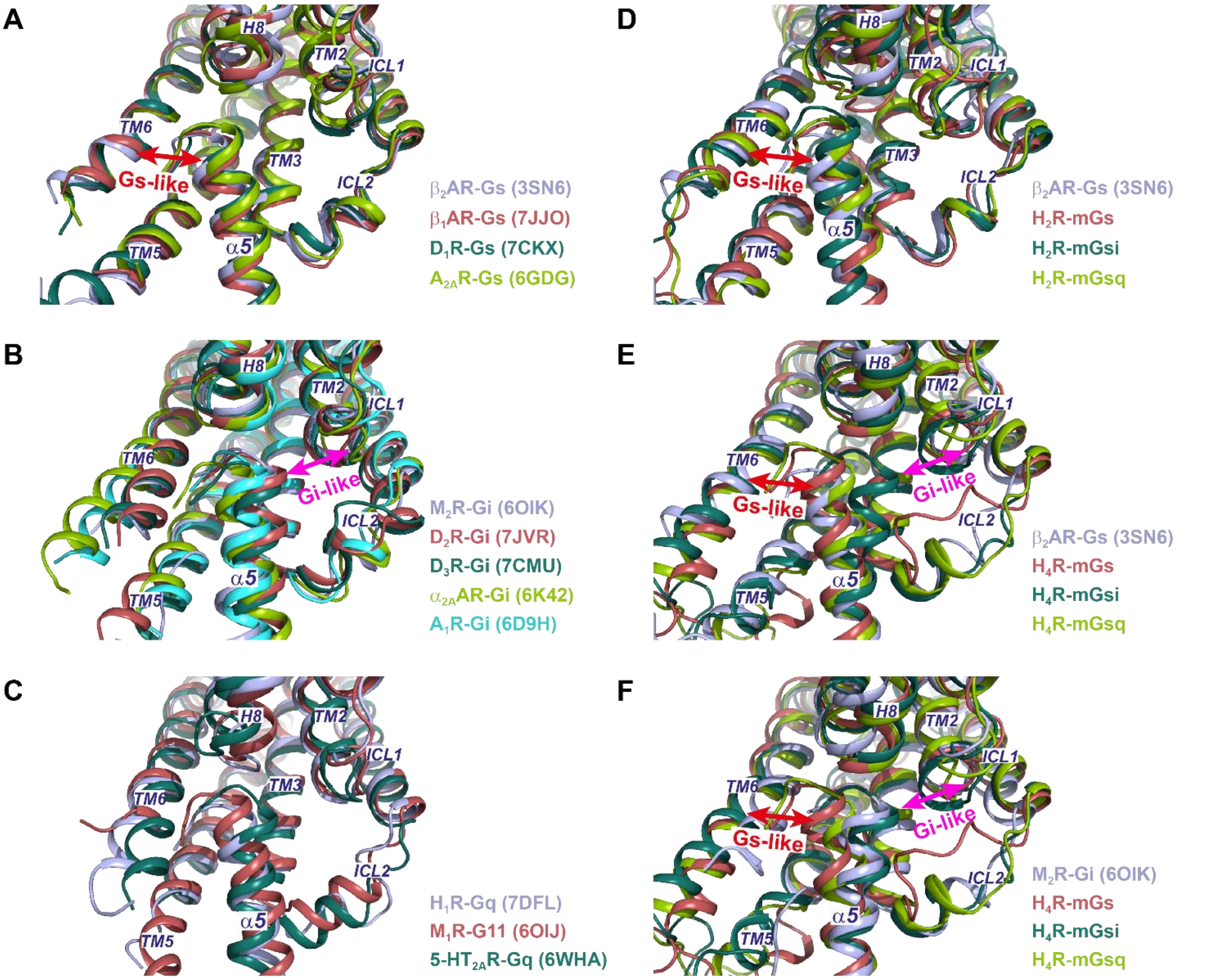

Figure S8. Comparison of the α5 helix orientation at the GPCR- G protein interface. Cytoplasmatic view of the α5 helix orientation of exemplary A) GPCR-Gs complexes (β_2AR, pdb-id.: 3SN6, purple; β_1AR, pdb-id: 7JJO, light red; D_1R, pdb-id.: 7CKX, dark green; A_{2A}R, pdb-id.: 6GDG, light green), B) GPCR-Gi complexes (M_2R, pdb-id.: 6OIK, purple; D_2R, pdb-id.: 7JVR, light red; D_3R, pdb-id.: 7CMU, dark green; α_{2A}AR, pdb-id.: 6K42, light green; A_1R, pdb-id.: 6DH9, blue) and GPCR-Gq complexes (H_1R, pdb-id.: 7DFL, purple; M_1R, pdb-id.: 6OIJ, light red; 5-HT_{2A}R, pdb-id.: 6WHA, dark green). D) Comparison of the α5 helix orientation in the β_2AR-Gs complex (purple) and the representative structures of low energy wells containing the fully active receptors bound to histamine ("B" states, cf. Fig. 1) of the H_2R-mGs (light red), H_2R-mGsi (dark green) and H_2R-mGsq (light green) complexes obtained in GaMD simulations. The α5 helix orientation in representative histamine bound structures of H_4R-mGs ("I1" state, light red), H_4R-mGsi ("B" state, dark green) and H_4R-mGsq ("B" state, light green) complexes are compared to the E) β_2AR-Gs (purple) and F) M_2R-Gi (purple) complexes. The Gs-like α5 orientation towards TM6 is highlighted in red and the Gi-like α5 orientation towards TM2 in pink.

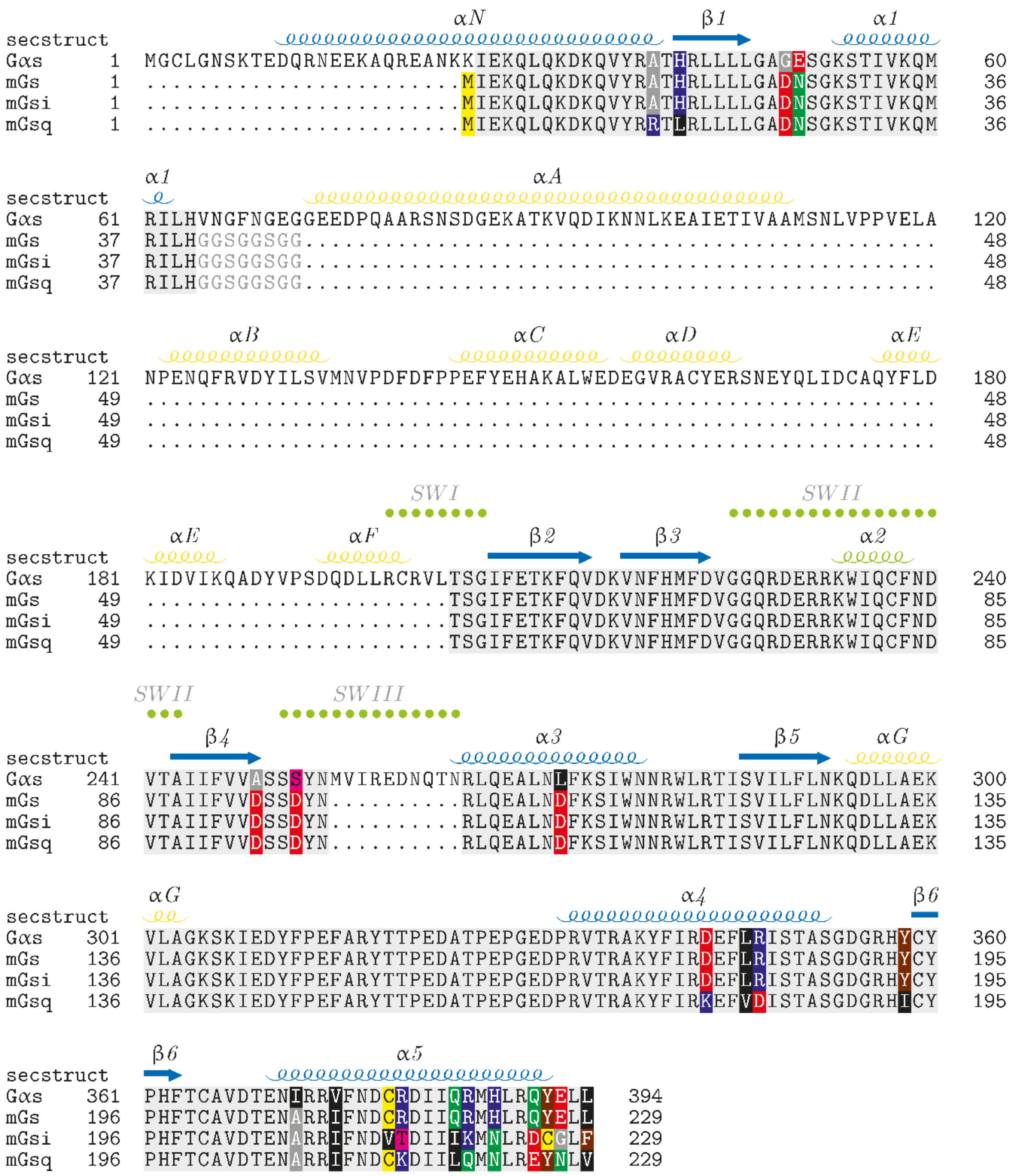

Figure S9. Sequence alignment of the Gαs subunit and the utilized mini-G proteins mGs, mGsi and mGsq. Secondary structure elements of Gαs, such as α helices and β sheets, are highlighted as loops and arrows, respectively, in blue (GTPase domain) and yellow (helical domain). The switch regions (SWI, SWII and SWIII) are labeled in green. Identical residues of the sequences are colored in light grey. Sequence differences are highlighted due to the chemical properties of the residue functional groups (acidic: red, aliphatic: black, aliphatic (small): grey, amide: green, aromatic: brown, basic: blue, hydroxyl: pink, imino: orange, sulfur: yellow). Residues only present in Gαs are not shaded.
